# 2-(1*H*-1,2,3-Benzotriazol-1-yl)-*N*′-(2-chloro­benzyl­idene)acetohydrazide

**DOI:** 10.1107/S1600536810050440

**Published:** 2010-12-08

**Authors:** Guo-Fang He, Zhi-Qiang Shi

**Affiliations:** a Department of Materials Science and Chemical Engineering, Taishan University, 271021 Taian, Shandong, People’s Republic of China

## Abstract

In the title compound, C_15_H_12_ClN_5_O, the mean planes of the benzotriazole and chloro­phenyl fragments form a dihedral angle of 70.8 (1)°. In the crystal, mol­ecules are linked into infinite chains along the *a* axis by N—H⋯O hydrogen bonds. Weak inter­molecular C—H⋯N hydrogen bonds further link these chains into layers parallel to the *ab* plane. The crystal studied was a racemic twin.

## Related literature

For related structures, see: Shi *et al.* (2007*a* ▶,*b* ▶); Ji & Shi (2008 ▶).
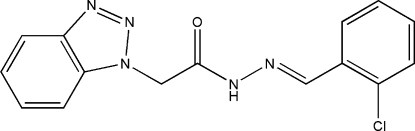


## Experimental

### 

#### Crystal data

C_15_H_12_ClN_5_O
*M* *_r_* = 313.75
Monoclinic, 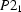

*a* = 4.6777 (16) Å
*b* = 11.726 (4) Å
*c* = 13.328 (5) Å
β = 94.224 (7)°
*V* = 729.0 (4) Å^3^
*Z* = 2
Mo *K*α radiation
μ = 0.27 mm^−1^
*T* = 295 K
0.12 × 0.10 × 0.08 mm

#### Data collection

Bruker APEXII CCD area-detector diffractometer
Absorption correction: multi-scan (*SADABS*; Bruker, 2005 ▶) *T*_min_ = 0.968, *T*_max_ = 0.979
3851 measured reflections
1902 independent reflections
1370 reflections with *I* > 2σ(*I*)
*R*_int_ = 0.039

#### Refinement

*R*[*F* ^2^ > 2σ(*F* ^2^)] = 0.043
*wR*(*F* ^2^) = 0.089
*S* = 1.01
1902 reflections
199 parameters
H-atom parameters constrained
Δρ_max_ = 0.16 e Å^−3^
Δρ_min_ = −0.17 e Å^−3^
Absolute structure: Flack (1983 ▶), 544 Flack pairs
Flack parameter: 0.55 (11)

### 

Data collection: *APEX2* (Bruker, 2005 ▶); cell refinement: *SAINT* (Bruker, 2005 ▶); data reduction: *SAINT*; program(s) used to solve structure: *SHELXTL* (Sheldrick, 2008 ▶); program(s) used to refine structure: *SHELXTL*; molecular graphics: *SHELXTL*; software used to prepare material for publication: *SHELXTL*.

## Supplementary Material

Crystal structure: contains datablocks global, I. DOI: 10.1107/S1600536810050440/cv5009sup1.cif

Structure factors: contains datablocks I. DOI: 10.1107/S1600536810050440/cv5009Isup2.hkl

Additional supplementary materials:  crystallographic information; 3D view; checkCIF report

## Figures and Tables

**Table 1:** Hydrogen-bond geometry (Å, °)

*D*—H⋯*A*	*D*—H	H⋯*A*	*D*⋯*A*	*D*—H⋯*A*
N4—H4⋯O1^i^	0.86	2.04	2.841 (4)	154
C7—H7*A*⋯N3^ii^	0.97	2.48	3.320 (6)	145

Symmetry codes: (i) 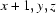; (ii) 
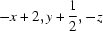
.

**Fig. 1.**
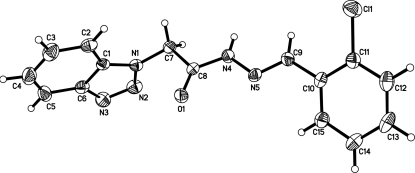
The molecular structure of (I), with displacement ellipsoids drawn at the 30% probability level.

**Fig. 2.**
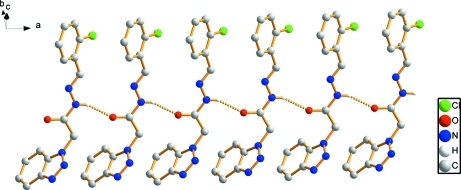
Hydrogen-bonded (dashed lines) chain in (I). H atoms not included in hydrogen bonding have been omitted for clarity.

## supplementary crystallographic information

### Comment

In continuation of our structural studies of benzotriazole derivatives (Shi *et al.*, 2007*a*,*b*; Ji *et al.*, 2008), herewith we present the crystal structure of the title compound, (I).

In (I) (Fig. 1), the bond lengths and angles are normal and in a good agreement with those observed in the related compounds (Shi *et al.*, 2007*a*,*b*; Ji *et al.*, 2008). The mean planes of the benzotriazole and chlorophenyl fragments form a dihedral angle of 70.8 (1)°.

In the crystal structure, the molecules are linked into infinite chains along the *a* axis by N—H···O hydrogen bonds (Table 1; Fig. 2). Weak intermolecular C—H···N hydrogen bonds (Table 1) link further these chains into layers parallel to *ab* plane.

### Experimental

The title compound was synthesized by the reaction of 2-(1*H*-1,2,3-benzotriazol-1-yl)acetohydrazide (1 mmol, 191.2 mg) with 2-chlorobenzaldehyde(1 mmol, 140.6 mg) in ethanol(20 ml)under reflux conditions (348 K) for 5 h. The solvent was removed and the solid product recrystallized from tetrahydrofuran. After five days colourless crystals suitable for X-ray diffraction study were obtained.

### Refinement

All H atoms were placed in idealized positions (C—H = 0.93— 0.97 Å, N—H = 0.86 Å) and refined as riding atoms. For those bound to C, *U*_iso_(H) = 1.2 or 1.5*U*_eq_(C). while for those bound to N, *U*_iso_(H) = 1.2 *U*_eq_(N).

### Crystal data

**Table:**

C_15_H_12_ClN_5_O	*F*(000) = 324
*M**_r_* = 313.75	*D*_x_ = 1.429 Mg m^−^^3^
Monoclinic, *P*2_1_	Mo *K*α radiation, λ = 0.71073 Å
Hall symbol: P 2yb	Cell parameters from 676 reflections
*a* = 4.6777 (16) Å	θ = 2.3–18.9°
*b* = 11.726 (4) Å	µ = 0.27 mm^−^^1^
*c* = 13.328 (5) Å	*T* = 295 K
β = 94.224 (7)°	Block, colourless
*V* = 729.0 (4) Å^3^	0.12 × 0.10 × 0.08 mm
*Z* = 2	

### Data collection

**Table:**

Bruker APEXII CCD area-detector diffractometer	1902 independent reflections
Radiation source: fine-focus sealed tube	1370 reflections with *I* > 2σ(*I*)
graphite	*R*_int_ = 0.039
phi and ω scans	θ_max_ = 25.0°, θ_min_ = 1.5°
Absorption correction: multi-scan (*SADABS*; Bruker, 2005)	*h* = −5→5
*T*_min_ = 0.968, *T*_max_ = 0.979	*k* = −9→13
3851 measured reflections	*l* = −15→14

### Refinement

**Table:**

Refinement on *F*^2^	Secondary atom site location: difference Fourier map
Least-squares matrix: full	Hydrogen site location: inferred from neighbouring sites
*R*[*F*^2^ > 2σ(*F*^2^)] = 0.043	H-atom parameters constrained
*wR*(*F*^2^) = 0.089	*w* = 1/[σ^2^(*F*_o_^2^) + (0.0267*P*)^2^ + 0.1806*P*] where *P* = (*F*_o_^2^ + 2*F*_c_^2^)/3
*S* = 1.01	(Δ/σ)_max_ < 0.001
1902 reflections	Δρ_max_ = 0.16 e Å^−^^3^
199 parameters	Δρ_min_ = −0.17 e Å^−^^3^
0 restraints	Absolute structure: Flack (1983), 544 Flack pairs
Primary atom site location: structure-invariant direct methods	Flack parameter: 0.55 (11)

### Special details

**Table:**

Geometry. All e.s.d.'s (except the e.s.d. in the dihedral angle between two l.s. planes) are estimated using the full covariance matrix. The cell e.s.d.'s are taken into account individually in the estimation of e.s.d.'s in distances, angles and torsion angles; correlations between e.s.d.'s in cell parameters are only used when they are defined by crystal symmetry. An approximate (isotropic) treatment of cell e.s.d.'s is used for estimating e.s.d.'s involving l.s. planes.
Refinement. Refinement of *F*^2^ against ALL reflections. The weighted *R*-factor *wR* and goodness of fit *S* are based on *F*^2^, conventional *R*-factors *R* are based on *F*, with *F* set to zero for negative *F*^2^. The threshold expression of *F*^2^ > σ(*F*^2^) is used only for calculating *R*-factors(gt) *etc*. and is not relevant to the choice of reflections for refinement. *R*-factors based on *F*^2^ are statistically about twice as large as those based on *F*, and *R*- factors based on ALL data will be even larger.

### Fractional atomic coordinates and isotropic or equivalent isotropic displacement parameters (Å^2^)

**Table:**

	*x*	*y*	*z*	*U*_iso_*/*U*_eq_	
Cl1	1.5224 (3)	0.95422 (11)	0.46424 (9)	0.0660 (4)	
O1	0.7318 (6)	0.5815 (3)	0.17718 (18)	0.0473 (8)	
N1	0.9309 (7)	0.4707 (3)	0.0172 (2)	0.0377 (8)	
N2	0.9247 (9)	0.3561 (3)	0.0325 (3)	0.0523 (10)	
N3	0.7297 (9)	0.3113 (3)	−0.0307 (3)	0.0561 (11)	
N4	1.1772 (7)	0.6360 (3)	0.2368 (2)	0.0413 (9)	
H4	1.3555	0.6412	0.2257	0.050*	
N5	1.0786 (8)	0.6779 (3)	0.3252 (2)	0.0391 (9)	
C1	0.7313 (9)	0.5002 (4)	−0.0583 (3)	0.0361 (10)	
C2	0.6509 (10)	0.6045 (4)	−0.1011 (3)	0.0466 (11)	
H2	0.7386	0.6726	−0.0805	0.056*	
C3	0.4329 (11)	0.6000 (5)	−0.1759 (3)	0.0619 (14)	
H3	0.3692	0.6677	−0.2063	0.074*	
C4	0.3028 (11)	0.4976 (6)	−0.2083 (3)	0.0632 (15)	
H4A	0.1586	0.4996	−0.2602	0.076*	
C5	0.3809 (10)	0.3947 (5)	−0.1661 (3)	0.0585 (14)	
H5	0.2931	0.3269	−0.1874	0.070*	
C6	0.6035 (10)	0.3978 (4)	−0.0881 (3)	0.0437 (11)	
C7	1.1297 (9)	0.5398 (4)	0.0782 (3)	0.0427 (11)	
H7A	1.1955	0.6024	0.0383	0.051*	
H7B	1.2950	0.4943	0.1011	0.051*	
C8	0.9886 (9)	0.5868 (4)	0.1684 (3)	0.0354 (9)	
C9	1.2636 (10)	0.7317 (3)	0.3822 (3)	0.0421 (11)	
H9	1.4451	0.7469	0.3612	0.050*	
C10	1.1839 (9)	0.7691 (4)	0.4815 (3)	0.0406 (10)	
C11	1.2939 (9)	0.8682 (4)	0.5275 (3)	0.0446 (11)	
C12	1.2219 (10)	0.8998 (4)	0.6219 (3)	0.0564 (13)	
H12	1.2975	0.9660	0.6516	0.068*	
C13	1.0391 (12)	0.8333 (5)	0.6715 (3)	0.0608 (15)	
H13	0.9924	0.8544	0.7355	0.073*	
C14	0.9219 (11)	0.7351 (4)	0.6283 (3)	0.0569 (14)	
H14	0.7953	0.6908	0.6623	0.068*	
C15	0.9967 (10)	0.7035 (4)	0.5332 (3)	0.0481 (12)	
H15	0.9198	0.6374	0.5038	0.058*	

### Atomic displacement parameters (Å^2^)

**Table:**

	*U*^11^	*U*^22^	*U*^33^	*U*^12^	*U*^13^	*U*^23^
Cl1	0.0682 (8)	0.0536 (7)	0.0772 (8)	−0.0105 (7)	0.0128 (7)	−0.0064 (7)
O1	0.0283 (18)	0.071 (2)	0.0436 (17)	−0.0008 (16)	0.0071 (13)	−0.0112 (15)
N1	0.042 (2)	0.032 (2)	0.0409 (19)	−0.0015 (18)	0.0082 (16)	−0.0021 (16)
N2	0.063 (3)	0.038 (2)	0.057 (2)	0.001 (2)	0.010 (2)	0.001 (2)
N3	0.071 (3)	0.038 (2)	0.059 (2)	−0.006 (2)	0.005 (2)	−0.006 (2)
N4	0.033 (2)	0.055 (2)	0.0374 (19)	0.0000 (18)	0.0100 (16)	−0.0138 (17)
N5	0.038 (2)	0.047 (2)	0.033 (2)	0.0031 (18)	0.0092 (17)	−0.0054 (17)
C1	0.035 (3)	0.039 (2)	0.036 (2)	0.000 (2)	0.0133 (19)	−0.0049 (19)
C2	0.051 (3)	0.041 (3)	0.049 (3)	0.003 (2)	0.014 (2)	0.002 (2)
C3	0.058 (4)	0.075 (4)	0.053 (3)	0.014 (3)	0.005 (3)	0.009 (3)
C4	0.051 (3)	0.096 (5)	0.042 (3)	0.007 (3)	0.004 (2)	−0.006 (3)
C5	0.051 (3)	0.079 (4)	0.046 (3)	−0.012 (3)	0.006 (2)	−0.020 (3)
C6	0.053 (3)	0.040 (3)	0.040 (2)	−0.002 (2)	0.014 (2)	−0.009 (2)
C7	0.043 (3)	0.052 (3)	0.035 (2)	−0.006 (2)	0.012 (2)	−0.009 (2)
C8	0.039 (3)	0.037 (2)	0.030 (2)	0.001 (2)	0.0049 (19)	0.0016 (18)
C9	0.041 (3)	0.046 (3)	0.040 (2)	−0.004 (2)	0.006 (2)	−0.007 (2)
C10	0.044 (3)	0.046 (3)	0.032 (2)	0.008 (2)	0.002 (2)	−0.0024 (19)
C11	0.044 (3)	0.046 (3)	0.044 (2)	0.004 (2)	0.002 (2)	−0.003 (2)
C12	0.067 (3)	0.054 (3)	0.048 (3)	0.008 (3)	−0.001 (2)	−0.016 (2)
C13	0.077 (4)	0.071 (4)	0.035 (3)	0.023 (3)	0.010 (3)	−0.007 (3)
C14	0.068 (4)	0.063 (3)	0.042 (3)	0.010 (3)	0.017 (2)	0.002 (2)
C15	0.051 (3)	0.049 (3)	0.044 (3)	0.003 (2)	0.005 (2)	−0.004 (2)

### Geometric parameters (Å, °)

**Table:**

Cl1—C11	1.733 (4)	C4—H4A	0.9300
O1—C8	1.217 (4)	C5—C6	1.416 (6)
N1—N2	1.359 (5)	C5—H5	0.9300
N1—C1	1.366 (5)	C7—C8	1.517 (5)
N1—C7	1.440 (5)	C7—H7A	0.9700
N2—N3	1.306 (5)	C7—H7B	0.9700
N3—C6	1.377 (5)	C9—C10	1.468 (5)
N4—C8	1.350 (5)	C9—H9	0.9300
N4—N5	1.387 (4)	C10—C15	1.386 (6)
N4—H4	0.8600	C10—C11	1.394 (5)
N5—C9	1.275 (5)	C11—C12	1.377 (5)
C1—C6	1.387 (5)	C12—C13	1.364 (6)
C1—C2	1.389 (6)	C12—H12	0.9300
C2—C3	1.374 (6)	C13—C14	1.383 (7)
C2—H2	0.9300	C13—H13	0.9300
C3—C4	1.399 (7)	C14—C15	1.390 (6)
C3—H3	0.9300	C14—H14	0.9300
C4—C5	1.370 (7)	C15—H15	0.9300
			
N2—N1—C1	109.9 (4)	C8—C7—H7A	109.5
N2—N1—C7	119.5 (4)	N1—C7—H7B	109.5
C1—N1—C7	130.6 (3)	C8—C7—H7B	109.5
N3—N2—N1	108.8 (4)	H7A—C7—H7B	108.1
N2—N3—C6	108.2 (3)	O1—C8—N4	123.9 (4)
C8—N4—N5	118.9 (3)	O1—C8—C7	123.2 (4)
C8—N4—H4	120.5	N4—C8—C7	112.9 (4)
N5—N4—H4	120.5	N5—C9—C10	118.5 (4)
C9—N5—N4	115.3 (3)	N5—C9—H9	120.7
N1—C1—C6	104.4 (3)	C10—C9—H9	120.7
N1—C1—C2	132.5 (4)	C15—C10—C11	118.0 (3)
C6—C1—C2	123.1 (4)	C15—C10—C9	119.6 (4)
C3—C2—C1	115.4 (5)	C11—C10—C9	122.4 (4)
C3—C2—H2	122.3	C12—C11—C10	121.3 (4)
C1—C2—H2	122.3	C12—C11—Cl1	119.3 (4)
C2—C3—C4	122.6 (5)	C10—C11—Cl1	119.5 (3)
C2—C3—H3	118.7	C13—C12—C11	119.6 (4)
C4—C3—H3	118.7	C13—C12—H12	120.2
C5—C4—C3	122.2 (5)	C11—C12—H12	120.2
C5—C4—H4A	118.9	C12—C13—C14	121.2 (4)
C3—C4—H4A	118.9	C12—C13—H13	119.4
C4—C5—C6	116.0 (5)	C14—C13—H13	119.4
C4—C5—H5	122.0	C13—C14—C15	118.8 (5)
C6—C5—H5	122.0	C13—C14—H14	120.6
N3—C6—C1	108.7 (4)	C15—C14—H14	120.6
N3—C6—C5	130.6 (4)	C10—C15—C14	121.1 (4)
C1—C6—C5	120.7 (4)	C10—C15—H15	119.4
N1—C7—C8	110.6 (3)	C14—C15—H15	119.4
N1—C7—H7A	109.5		
			
C1—N1—N2—N3	0.4 (4)	N2—N1—C7—C8	−93.6 (4)
C7—N1—N2—N3	179.9 (3)	C1—N1—C7—C8	85.8 (5)
N1—N2—N3—C6	−0.5 (5)	N5—N4—C8—O1	3.6 (6)
C8—N4—N5—C9	−174.3 (4)	N5—N4—C8—C7	−177.1 (3)
N2—N1—C1—C6	−0.1 (4)	N1—C7—C8—O1	−11.3 (6)
C7—N1—C1—C6	−179.6 (4)	N1—C7—C8—N4	169.5 (3)
N2—N1—C1—C2	179.1 (4)	N4—N5—C9—C10	−174.2 (3)
C7—N1—C1—C2	−0.4 (7)	N5—C9—C10—C15	33.0 (6)
N1—C1—C2—C3	−178.9 (4)	N5—C9—C10—C11	−148.1 (4)
C6—C1—C2—C3	0.2 (6)	C15—C10—C11—C12	0.7 (6)
C1—C2—C3—C4	−1.0 (6)	C9—C10—C11—C12	−178.2 (4)
C2—C3—C4—C5	1.2 (8)	C15—C10—C11—Cl1	−178.6 (3)
C3—C4—C5—C6	−0.5 (7)	C9—C10—C11—Cl1	2.6 (6)
N2—N3—C6—C1	0.5 (5)	C10—C11—C12—C13	−0.2 (7)
N2—N3—C6—C5	−179.5 (4)	Cl1—C11—C12—C13	179.1 (4)
N1—C1—C6—N3	−0.2 (4)	C11—C12—C13—C14	−0.7 (7)
C2—C1—C6—N3	−179.5 (4)	C12—C13—C14—C15	0.9 (7)
N1—C1—C6—C5	179.7 (3)	C11—C10—C15—C14	−0.4 (6)
C2—C1—C6—C5	0.4 (6)	C9—C10—C15—C14	178.5 (4)
C4—C5—C6—N3	179.7 (5)	C13—C14—C15—C10	−0.4 (7)
C4—C5—C6—C1	−0.2 (6)		

### Hydrogen-bond geometry (Å, °)

**Table:**

*D*—H···*A*	*D*—H	H···*A*	*D*···*A*	*D*—H···*A*
N4—H4···O1^i^	0.86	2.04	2.841 (4)	154.
C7—H7A···N3^ii^	0.97	2.48	3.320 (6)	145.

Symmetry codes: (i) *x*+1, *y*, *z*; (ii) −*x*+2, *y*+1/2, −*z*.
